# Coexistence of papillary thyroid carcinoma in secondary hyperparathyroidism

**DOI:** 10.1186/s12893-021-01330-z

**Published:** 2021-09-06

**Authors:** Junhao Ma, Zhuochao Mao, Yunjin Yao, Yimin Lu, Haohao Wang, Yan Yang, Jun Yang, Weibin Wang, Lisong Teng

**Affiliations:** 1 Department of Surgical Oncology, First Affiliated Hospital, Zhejiang University, Zhejiang, China; 2 Department of Nuclear Medicine, First Affiliated Hospital, Zhejiang University, Zhejiang, China; 3 Department of Laboratory, First Affiliated Hospital, School of Medicine, Zhejiang, China

**Keywords:** Papillary thyroid carcinoma, Secondary hyperparathyroidism, Tumor characteristics, Morbidity, Occult papillary thyroid carcinoma, Retrospective case–control study

## Abstract

**Background:**

The coexistence of primary hyperparathyroidism and papillary thyroid carcinoma (PTC) is common and may be associative with more aggressive PTC, with higher rates of extrathyroidal extension and multicentricity. However, it is unclear whether secondary hyperparathyroidism (SHPT) is associated with more invasive PTC in terms of morbidity, tumor pathological characteristics, and prognosis. The aim of this study was to evaluate the rate and tumor characteristics of PTC in patients with SHPT.

**Methods:**

A total of 531 patients diagnosed with SHPT who underwent surgery from August 2013 to December 2018 at the First Affiliated Hospital of Zhejiang University were evaluated retrospectively. Patient demographics, surgical records, and follow-up information were recorded and analyzed. Control subjects were matched to the enrolled patients in a 1:4 ratio in terms of age, sex and pathological subtype.

**Results:**

Among the 531 patients with SHPT who underwent surgery, 34 had coexisting PTC and PTC + SHPT (6.4%). The mean tumor diameter in the PTC + SHPT group was smaller than that in the PTC group (5.57 mm vs 9.00 mm, *p* < 0.001). The proportion of papillary thyroid micro-carcinoma in the PTC + SHPT group was significantly higher than that in the PTC group (29 [85.29%] vs. 86[63.24%], *p* = 0.014). There were no statistically significant differences between groups in terms of tumor multicentricity (15 [44.12%] vs 39 [28.68%], *p* = 0.066), tumor bilaterality (9 [26.47%] vs. 29 [21.32%], *p* = 0.499), tumor extrathyroidal extension (2 [5.88%] vs. 19 [13.97%], *p* = 0.255), or lymph node (LN) metastasis rate (12 [35.29%] vs. 49 [36.03%], *p* = 1.000). However, the PTC + SHPT and PTC groups were significantly different in terms of contralateral thyroidectomy (10 [29.41%] vs. 70 [51.47%], *p* = 0.023) and lymph node dissection (22 [64.71%] vs. 125 [91.91%], *p* < 0.001).There was no significant difference between the PTC + SHPT and PTC groups in terms of prognostic staging (33 [97.06%] vs. 122 [89.71%], *p* = 0.309) or recurrence (mean follow-up time: 36 months vs. 39 months, *p* = 0.33).

**Conclusions:**

The prevalence of PTC is high in patients with SHPT; compared with PTC in the general population, most papillary thyroid carcinomas with SHPT are occult thyroid carcinomas and present no significant difference in terms of tumor pathological features and prognostic staging. It is necessary for surgeons to perform more adequate preoperative examination and be more careful during surgery to avoid missing the coexistence of PTC in patients with SHPT.

## Background

Secondary hyperparathyroidism, a common complication of end-stage renal disease (ESRD), may ultimately develop in nearly all patients with chronic kidney disease [1, 2].It is responsible for bone pain, itching, mineral bone disorders and ESRD and is associated with a high risk of cardiovascular events and death [3, 4]. Some patients with early-stage SHPT can be treated with drugs such as lanthanum carbonate and cinacalcet; however, surgical intervention is required in advance stages of the disease as drug therapy is ineffective or parathyroid hormone (PTH) level increase above a certain range [5–7].

As the most common thyroid carcinoma (TC), PTC usually presents as an indolent tumor with high incidence and low mortality [8, 9]. A high incidence of TC in patients with ESRD has led to growing interest in investigation of the impact of SHPT on TC in terms of occurrence and tumor biological behavior [10]. However, it is unclear whether SHPT is associated with more invasive PTC in terms of morbidity, tumor pathological characteristics, and prognosis. Therefore, this study aimed to evaluate the rate and tumor characteristics of PTC in patients with SHPT.

## Methods

### Patients and methods

A total of 531 patients with SHPT who underwent surgery from August 2013 to December 2018 at the First Affiliated Hospital of the Zhejiang University were evaluated retrospectively. Patient demographics, operation records and follow-up information were recorded and analyzed. Patients with primary hyperparathyroidism, tertiary hyperparathyroidism or multiple endocrine neoplasms were excluded. Control subjects were matched to the enrolled patients 1:4 ratio in terms of age, sex, and pathological subtype. The study protocol was approved by the Ethics Committee of the First Affiliated Hospital of Zhejiang University.

### Surgical indication and approach

Patients with SHPT were surgically treated according to the recommendations of the latest guidelines of Kidney Disease: Improving Global Outcomes 2009: In patients with CKD stage 5D and elevated or rising PTH, we suggest calcitriol, or vitamin D and analogs, or calcimimetics, or a combination of calcimimetics and calcitriol or vitamin D analogs be used to lower PTH. In patients with CKD 3-5D with severe hyperparathyroidism (HPT) who fail to respond to medical/pharmacological therapy, we suggest parathyroidectomy [11]. Among 34 patients with PTC + SHPT, 33 patients underwent total parathyroidectomy with auto-transplantation, and one patient underwent subtotal parathyroidectomy. In the 136 patients with PTC, thyroidectomy indications and surgical procedures followed the latest guidelines for PTC of the American Thyroid Association [12]. Total thyroidectomy was performed in patients with bilateral tumors, multiple tumors, abnormal lymph nodes, or extrathyroidal extension based on preoperative examination or intraoperative evaluation findings. Central neck dissection (CND) included removal of all nodes and fibro-fatty tissue extending vertically from the hyoid bone to the thoracic inlet and laterally from the medial border of the common carotid artery to the midline of the trachea. Therapeutic CND was performed if abnormal lymph nodes were detected on preoperative or intraoperative examination; prophylactic CND was considered for tumors with T stage T3 and T4. Lateral neck dissection (LND), including modified radical neck dissection and selective neck dissection, was performed when lateral LN metastasis was confirmed preoperatively [13].

### Statistical analysis

Statistical analyses were performed using SPSS software (version 22.0; IBM Corp., Armonk, NY, USA). Descriptive statistics for continuous variables are expressed as mean ± standard deviation, and non-normally distributed variables are expressed as median (min–max). Bivariate analysis was conducted with independent sample t-test, to compare means. Categorical variables are expressed as number and percentage, and Fisher’s Chi-square test was used to assess the differences between groups with regard to categorical variables. Differences were considered statistically significant at *p* < 0.05.

## Results

### PTC + SHPT screening process

**Fig. 1:**
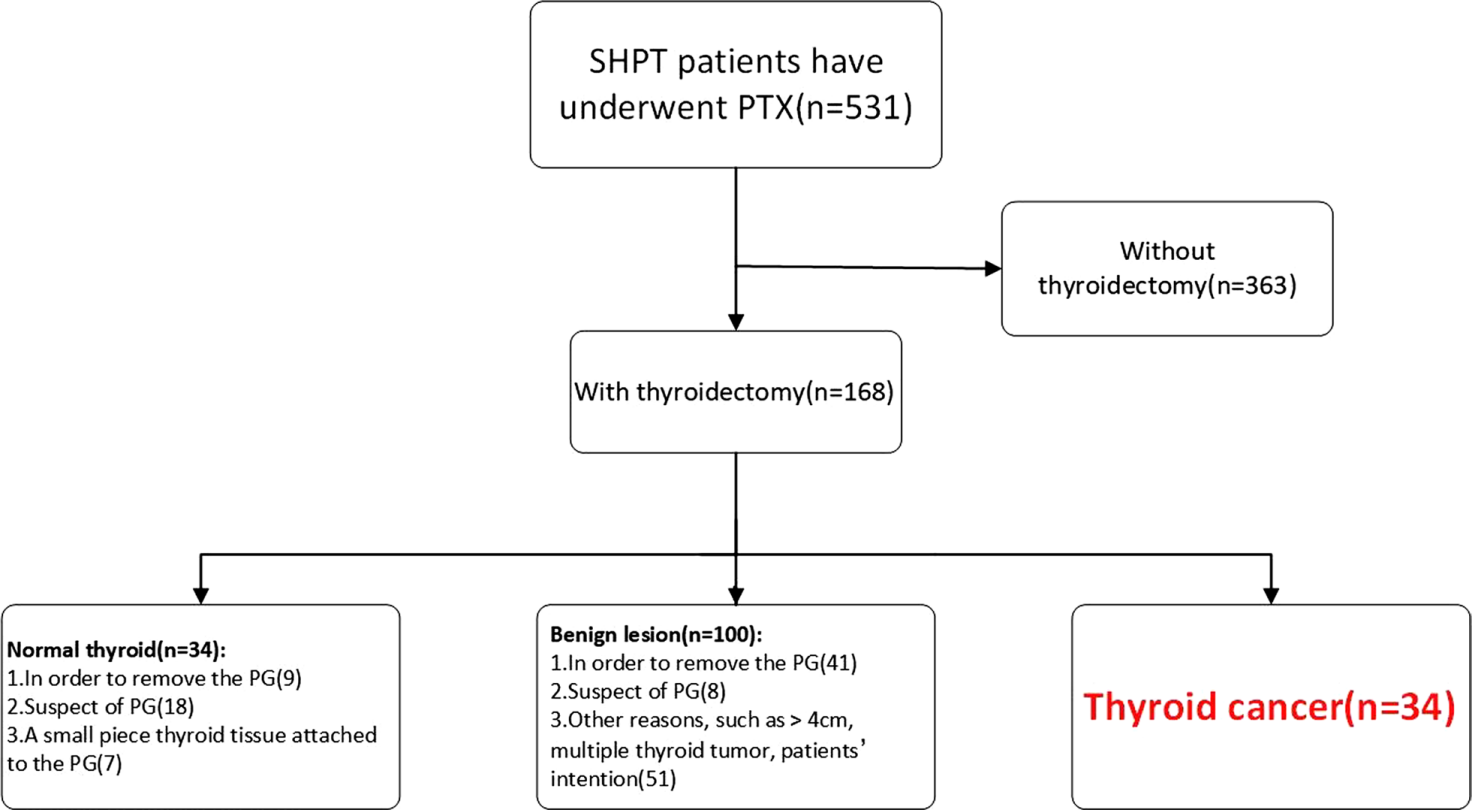
Screening process of PTC + SHPT among patients with SHPT. *PTC* papillary thyroid carcinoma, *SHPT* secondary hyperparathyroidism, *PTX* parathyroidectomy, *PG* parathyroid gland

All 531 patients with SHPT underwent parathyroidectomy. Of these patients, 168 patients also underwent thyroidectomy. Based on the pathological diagnosis of the removed thyroid tissue, patients were divided into three subgroups: patients with normal thyroid tissue, patients with benign thyroid nodules and patients with thyroid carcinoma (Fig. 1). Among the 34 patients with normal thyroid tissue (first subgroup), nine patients underwent thyroidectomy because the abnormal anatomy of the thyroid glands obstructed resection of the parathyroid gland (PG); 18 patients underwent thyroidectomy because the normal thyroid tissues were mistaken for the PG, and seven underwent thyroidectomy had a small piece of thyroid tissue attached to the resected PG. Among the 100 patients with benign thyroid nodules (second subgroup), 41 patients underwent thyroidectomy because the anatomy of the thyroid gland obstructed resection of PG; eight patients underwent thyroidectomy because the normal thyroid tissues were mistaken for the PG, and 51 patients underwent thyroidectomy due to other reasons (such as tumor diameter > 4 cm, multiple thyroid nodules, and patients’ intentions). In the third subgroup, all 34 cases had TC, pathologically proved to be PTC; the incidence of PTC in the group of patients with SHPT who required surgery was 6.4% (34/531). Among them, 17 patients were preoperatively suspected to have cancer on ultrasonography. However, another 17 patients who were not preoperatively suspected to have malignancy were diagnosed with PTC based on intraoperative frozen section or postoperative pathological findings.

### Clinical characteristics of patients with PTC + SHPT

Of the 34 patients with PTC + SHPT, 15 patients were men, and the average age was 47 years. All patients had stage 5 chronic kidney disease combined with SHPT. The average preoperative parathyroid hormone level was 2010 pg/mL, and the average postoperative hormone level was 24 pg/mL (Table 1).

**Table 1:** Basic information of the PTC + SHPT group

	PTC + SHPT(n = 34)
Male	15 (44.1%)
Age* (years)	47 (30–69)
Serum iPTH pre-operative* (pg/ml)	2010 (203–4133)
Serum iPTH pro-operative* (pg/ml)	24 (3–392)

*PTC* papillary thyroid carcinoma, *SHPT* secondary hyperparathyroidism

*Median (min–max)

### Comparison of tumor characteristics between the PTC + SHPT and PTC groups

A total of 136 patients with PTC were selected as the control group after matching to the enrolled patients in a 1:4 ratio in terms of age, sex, and pathological subtype. The comparison of tumor characteristics between the PTC + SHPT and PTC groups is shown in Table 2. The mean tumor diameter in the PTC + SHPT group was smaller than that in the PTC group (5.57 mm vs 9.00 mm, *p* < 0.001). Most tumors were papillary thyroid microcarcinomas (PTMCs), and the proportion of papillary thyroid microcarcinoma (PTMC) in the PTC + SHPT group was significantly higher than that in the PTC group (29 [85.29%] vs. 86 [63.24%], *p* = 0.014). However, there was no statistically significant difference between groups in term of tumor multicentricity (15 [44.12%] vs. 39 [28.68%], *p* = 0.066), tumor bilaterality (9 [26.47%] vs. 29 [21.32%], *p* = 0.499), tumor extrathyroidal extension (2 [5.88%] vs. 19 [13.97%], *p* = 0.255) and lymph node metastasizes rate (12 [35.29%] vs. 49 [36.03%], *p* = 1.000).

**Table 2:** Comparison of tumor characteristics between the PTC + SHPT and PTC groups

	PTC + SHPT (n = 34)	PTC (n = 136)	*p*
Age* (years)	47 (30–69)	47 (30–69)	1.000
Male	15 (44.1%)	15 (44.1%)	1.000
Tumor diameter* (mm)	5.57 (0.5–15)	9.00 (0.5–40)	< 0.001
Tumor diameter < 10 mm	29 (85.29%)	86 (63.24%)	0.014
Tumor bilaterality	9 (26.47%)	29 (21.32%)	0.499
Tumor multicentricity	15 (44.12%)	39 (28.68%)	0.066
Tumor extrathyroidal extension	2 (5.88%)	19 (13.97%)	0.255
Lymph node metastasis rate	12 (35.29%)	49 (36.03%)	1.000

*PTC* papillary thyroid carcinoma, *SHPT* secondary hyperparathyroidism

*p* < 0.05 was considered statistically significant

*Average (min–max)

### Comparison of surgical approach between the PTC + SHPT and PTC groups

A comparison of the surgical approach between the PTC + SHPT and PTC groups is shown in Table 3. Contralateral thyroidectomy, included contralateral partial thyroidectomy, contralateral subtotal thyroidectomy and contralateral total thyroidectomy; lymph node dissection, included CND and LND. The percentage of contralateral thyroidectomy (10 [29.41%] vs. 70 [51.47%], *p* = 0.023) and lymph node dissection (22 [64.71%] vs. 125 [91.91%], *p* < 0.001) during surgery were significantly lower than in the PTC + SHPT group than in the in PTC group.

**Table 3:** Comparison of surgical approach between the PTC + SHPT and PTC groups

	PTC + SHPT (n = 34)	PTC (n = 136)	*p*
Contralateral thyroidectomy	10 (29.41%)	70 (51.47%)	0.023
Lymph node dissection	22 (64.71%)	125 (91.91%)	< 0.001

*PTC* papillary thyroid carcinoma, *SHPT* secondary hyperparathyroidism

*p* < 0.05 was considered statistically significant

### Prognostic staging and survival in the PTC + SHPT and PTC groups

**Fig. 2:**
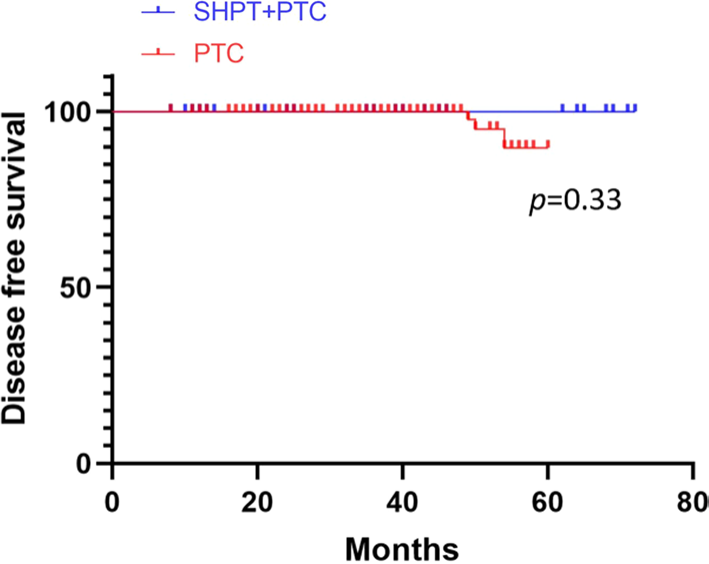
Disease free survival of the PTC + SHPT and PTC groups

All patients with PTC in our study had stage I or II disease, as shown in Table 4. There was no statistical significance between the PTC + SHPT and PTC groups (33 [97.06%] vs. 122 [89.71%], *p* = 0.309). The mean follow-up time for the 34 patients with PTC + SHPT was 36 months. Among them, one patient was lost to follow-up, one patient died of heart failure, and 32 patients had no recurrence by the end of the follow-up. The mean follow-up time for the 136 patients with PTC was 39 months. Among them, one patient was lost to follow-up, no patients died, three patients experienced relapse, and 132 patients had no recurrence by the end of the follow-up. The disease-free survival rates of the two groups are shown in Fig. 2, *p* = 0.33.

**Table 4:** Comparison of prognostic staging between the PTC + SHPT and PTC groups

Prognostic staging	PTC + SHPT (n = 34)	PTC (n = 136)	*p*
I	33 (97.06%)	122 (89.71%)	0.309
II	1 (2.94%)	14 (10.29%)	0.309

*PTC* papillary thyroid carcinoma, *SHPT* secondary hyperparathyroidism

*p* < 0.05 was considered statistically significant

## Discussion

In this study, we analyzed 34 patients with SHPT and pathologically proven PTC from 531 patients with SHPT who underwent surgery to investigate whether SHPT is associated with more aggressive PTC in terms of morbidity, tumor pathological characteristics and prognosis.

According to previous research, several factors such as metabolic disorders of calcium, phosphorus, and vitamin D induced by SHPT; immunological incompetence accompanied by ESRD; and aging have been suggested to be involved in the occurrence and development of thyroid dysfunction and PTC [14, 15]. Lin et al. found that ESRD patients with SHPT exhibited a 10.1-fold increased risk of TC compared to ESRD patients without SHPT [16]. However, another study investigated 339 ESRD patients with SHPT who underwent surgical therapy and showed that the incidence of PTC was 2.4% in the SHPT group [17]. Compared with the PTC detection rate in autopsy which ranged from 5 to 11%, no significant correlation was found between PTC and SHPT. Therefore, it is still debated whether ESRD or SHPT can induce a higher incidence rate of PTC. Our study showed that the incidence of PTC in the group of patients with SHPT who needed surgery was 6.4% (34/531), higher than the incidence of PTC in the general population (0.5–6.9/100,000) in China [18]. Therefore, we believe that SHPT is a risk factor for PTC.

As previously shown, both sex and age are associated with the development of PTC [19]. The PTC + SHPT group included 34 patients (44.1% men and 55.9% women), with an average age of 47 years. We then matched the control group to the patient group in terms of age, sex and pathological subtype in a 1:4 ratio. Compared to the PTC group, the PTC + SHPT group had a smaller tumor diameter, which is the main characteristic of occult thyroid carcinoma. However, there were no statistically significant differences between groups in terms of tumor laterality (unilateral or bilateral), tumor multicentricity, tumor extra-thyroid extension, lymph node metastasis rate, or prognostic staging. Among the 34 patients in the PTC + SHPT group, 12 (35.29%) patients had lymph node metastasis. Additionally, among the 29 patients with PTMC without SHPT, nine (31.03%) patients had lymph node metastasis, in accordance with previously reported data [13]. From the above results, we could not find any associative etiology of SHPT with more aggressive PTC in the PTC + SHPT group. In contrast, the tumor characteristics of PTC in the PTC + SHPT group were similar to those of occult PTC, mostly composed of PTMC (85.29%). Reviewing the preoperative ultrasonography and surgical records of 34 patients with PTC, we found that 17 patients were not suspected to have TC preoperatively, but underwent thyroidectomy because of suspected thyroid carcinoma during surgery. These factors led to an increased detection rate of occult thyroid carcinoma which tends to be smaller in diameter.

When we performed PTC surgery during parathyroidectomy, there was a smaller chance of contralateral thyroidectomy or lymph node dissection. The above data suggested that papillary thyroid carcinomas in the PTC + SHPT group were often occult PTC, presenting a more indolent tumor phenotype associated with minimal tissue trauma and maximal thyroid function retention. It has previously been proven that PTC, an endocrine tumor, has good prognosis, with very low mortality [20, 21]. A previous study has shown that the relapse rate of PTC is approximately 1–5% [22]. The follow-up data of the two groups in our study showed that there was no recurrence in the PTC + SHPT group, three patients had recurrence in the PTC group. Base on the above prognostic data, we can conclude that SHPT is not a risk factor for PTC recurrence. Based on our study, we can conclude that PTC has a higher incidence in surgically treated patients with SHPT and that occult PTC is more common.

Nevertheless, this study has several limitations. The sample size of the study was limited, and the follow-up time was short. Therefore, more prospective randomized controlled studies with larger sample sizes and longer follow-up times are needed to further validate the study conclusions.

## Conclusions

In conclusion, the prevalence of PTC is high in patients with SHPT; thus clinicians must be aware for the coexistence of PTC during surgery. Compared with PTC in the general population, most of papillary thyroid carcinomas with SHPT are occult thyroid carcinomas and present no significant differences in terms of tumor pathological features and prognostic staging. It is necessary for surgeons to perform more adequate preoperative examination and be more careful during the surgery to avoid missing the coexistence of PTC in patients with SHPT.

## Data Availability

The datasets used and analyzed during the current study are available from the corresponding author on reasonable request.

## Abbreviations

PTC: Papillary thyroid carcinoma
TC: Thyroid carcinoma
SHPT: Secondary hyperparathyroidism
PTMC: Papillary thyroid microcarcinoma
PG: Parathyroid gland
ESRD: End-stage renal disease
PTH: Parathyroid hormone
CND: Central neck dissection
LND: Lateral neck dissection
LN: Lymph node
